# Why are they eco-friendly? Attributing eco-friendly descriptive norms to intrinsic motivation increases pro-environmental purchase intention

**DOI:** 10.1371/journal.pone.0265839

**Published:** 2022-10-20

**Authors:** Emma Ejelöv, Magnus Bergquist, André Hansla, Andreas Nilsson

**Affiliations:** 1 Department of Psychology, University of Gothenburg, Gothenburg, Sweden; 2 Centre for Collective Action Research, University of Gothenburg, Gothenburg, Sweden; Kyonggi University, REPUBLIC OF KOREA

## Abstract

People perform pro-environmental behaviors not only out of intrinsic motivation, but also due to external factors such as expected social approval or financial gain. To the extent that people use their own motivations to infer the motivation of others, people may view descriptive norms favoring pro-environmental behavior as extrinsically motivated. This may in turn decrease the normative influence of the norm, as conformity can be negatively affected by perceptions that others are conforming mindlessly. While descriptive norms generally promote pro-environmental behavior change, the influential power of descriptive norms varies between studies. One possible explanation for these inconclusive findings is that people interpret others’ behavior as either intrinsically- or extrinsically motivated. We propose that pro-environmental descriptive norms will be more influential when attributing others’ pro-environmental behavior as intrinsically (e.g., pleasure of contributing to the environment) rather than extrinsically, motivated (e.g., fear of social disapproval). In two experiments (*N* = 1326), we compared participants’ intention to purchase pro-environmental products between four conditions: control condition vs intrinsic norm vs extrinsic norm (Exp. 1) vs injunctive norm (Exp. 2). Results consistently found a significant increase in pro-environmental purchase intention in the intrinsic norm condition compared to both extrinsic norm condition (Exp. 2) and no-information control condition (Exp. 1 & 2). These studies highlight that attribution of behavior is vital for the adoption of pro-environmental norms.

## Introduction

Imagine the following: you are in a grocery store and are about to decide which one of two similar products to buy, when you notice most people around you are choosing product X. While you neither know, nor have the time to consider their reasons, steered by a descriptive social norm you may end up conforming to their behavior without too much reflection. But what if you had considered or even known their reasons for choosing that product beforehand? Let’s say you found out other people chose product X *because* they were all deeply concerned for the environment, or *because* it was the cheapest option. Would you then have been more or less inclined to follow their choice? In other words, does attributing an observed normative behavior to some salient internal or external cause matter for normative influence? If so, how? These questions are important because many situations contain cues of external incentives. For example, if there is a discount on a specific green product, we may assume that others buy this product because of a financial incentive. If this is the case, we may refrain from buying other, more expensive, green products, because we do not believe that others purchase green products for their environmental benefit. If these external incentives cause people to assume that others are extrinsically motivated, we need to know how this affects conformity levels, as it could potentially decrease the effectiveness of social norm interventions. The present research therefore addresses these questions. Specifically, we test and support the novel hypothesis that people are more likely to conform to others’ pro-environmental choice if others actually care about the environment rather than act pro-environmental simply because they fear others’ social disapproval. Hence, we incorporate research on intrinsic vs extrinsic motivation [1] to gain better understanding of when people actually conform to pro-environmental descriptive norms.

A bulk of experimental research now shows that social norm interventions, typically targeting descriptive norms (i.e., highlighting what other people are doing) and/or injunctive norms (i.e., highlighting what behavior other people approve/disapprove of), can successfully increase a range of pro-environmental behaviors [2–7]. However, there is meta-analytic evidence suggesting that the effectiveness of social norm interventions varies considerably, with some interventions even showing that social norms can have negative effects [3]. This suggests that there are important moderators of social norms to consider when designing interventions. While interventions have mainly targeted descriptive and injunctive norms, correlational studies have generally highlighted the importance of personal norms for promoting pro-environmental behavior [8–10]. Compared to social norms, such norms may in fact be an even stronger predictor of pro-environmental behavior [11]. A feature of personal norms that may explain why they guide behavior to a larger extent is that personal norms are more internalized than are injunctive and descriptive norms, in the sense that the motivation to conform to the norm originates from the individual’s intrinsic motivation, such as values and beliefs, rather than extrinsic motivation, such as expectations of external sanctions [11–13]. While intrinsic motivation may be a better predictor of pro-environmental behavior, research suggests that many people in fact perform pro-environmental behaviors due to external factors. Analysis of verbal explanations of pro-environmental behaviors have shown that only around 5% tended to describe behavior in terms of internal beliefs [14]. If people also perceive that others in fact are performing pro-environmental behaviors due to extrinsic motivation, this could potentially decrease the tendency to conform to the behavior of others, as there is research to suggest that conformity to minority norms increases when the minority communicates a strong internal conviction [15], while conformity decreases when others are perceived as mindlessly conforming [16]. Given that people engage in spontaneous attributions of behavior, we are potentially missing an important contextual moderator of normative influence when we lack knowledge on how attributional tendencies affect conformity to social norms.

Drawing on research suggesting that intrinsic motivation is generally more influential than extrinsic motivation in terms of exhibiting behavioral control and consistency to achieve a valued outcome [1, 17, 18], in this article, we examine whether and how intrinsic vs extrinsic attributions of conformity would moderate the influence of social norms. Specifically, we test if individuals are more inclined, as we expect them to be, to conform with a pro-environmental behavior (here a green consumer choice serving as a descriptive norm) when they attribute it to intrinsic rather than extrinsic causes. In addition, we test if such an effect would “spill over” and influence also some non-targeted pro-environmental purchase intentions.

### Motivational underpinnings of norms

Social norms are often defined as ‘rules and standards that are understood by members of a group, and that guide and/or constrain behavior’ [19], and can be characterized as the perceived behavioral pattern and/or (dis)approval of others [20]. This definition emphasizes a distinction made between descriptive norms and injunctive norms, with descriptive norms conveying what other people are doing and injunctive norms conveying what other people approve or disapprove of [20]. Conformity to social norms typically increases when these two types of norms are aligned, i.e., when other people do (descriptive norm) what others approve of (injunctive norms) or vice versa [4, 6, 7]. These two types of norms may be said to correspond to different motivational underpinnings, with conformity to descriptive norms being motivated by a desire to act correctly/effectively, and conformity to injunctive norms being motivated by a need to gain (avoid) social (dis)approval [20, 21]. Adding to these sources of motivation, personal norms are assumed to drive behavior due to an understanding that compliance will result in a valued outcome [11, 22, 23].

Motivation to conform to norms can also be conceptually distinguished based on the norm’s level of internalization [24], with personal norms being more internalized than descriptive and injunctive norms [11]. This division of norms according to their level of internalization overlaps with, and is partly based on, Deci and Ryan’s [25, 26] work on intrinsic and extrinsic motivation in the self-determination theory (SDT). Personal norms are classified as a more internalized norm because it is expected to be self-determined, in that the motivation to conform to the norm originates from the individuals’ moral standard, and thus is not socially (or, externally) reinforced, as for example injunctive norms are [11, 13]. In SDT, internalized motivation is denoted as intrinsic motivation, and refers to engaging in behaviors because of the innate pleasure and satisfaction a person derives from conducting the behavior. Although the original definition of intrinsic motivation is mainly enjoyment-based, normative considerations have been proposed in the definition, for example feelings of obligation and acting out of principle [27, 28]. Extrinsic motivation on the other hand is an external and more instrumental type of motivation, where behavior is performed in order to achieve rewards or avoid punishments that originates from the person’s environment (e.g., other people) [29]. Intrinsic motivation can however be undermined by the presence of external incentives, presumably because such rewards shifts a person’s locus of control to an external source [30–32]. These two motivations differ in the degree to which the value and importance of the behavior in question has been internalized, where behavior that is intrinsically motivated is perceived as more important to the person than if the behavior had been extrinsically motivated [17]. Presumably for this reason, intrinsic motivation typically correlates with pro-environmental behavior to a greater extent than extrinsic motivation [33–36].

Thus, intrinsic motivation and internalized norms predict pro-environmental behavior to a greater extent than extrinsic motivation and externalized norms. Norm constructs and their motivational influence have previously been discussed in the context of their level of internalization, that is, to the extent that their reinforcing effects originate within (e.g., in the form of values or felt obligation) or outside (e.g., in the form of social disapproval) of the individual [11, 24]. However, research has not focused on *why* people think others are conforming to a norm. First, what is the perceived motivational basis for other people’s behavior, and second, how do these motives affect individual’s tendency to conform?

### Attribution of social norms

To the extent that normative influence is not a strictly heuristic process, is other people’s conformity to social norms likely to be attributed to intrinsic or extrinsic motivation? This may depend on whether or not social norms are perceived as an external motivator. While research on the correspondence bias show that people have a general tendency to attribute other peoples’ behavior internally [37] and the actor-observer bias that people tend to attribute their own behavior to situational factors, research suggests that the presence of external incentives can alter this bias. Self-perception theory [38] posits that people use other peoples’ behavior to infer the personal attitudes of others. When there is no obvious external motivator for a person’s behavior as judged by the context, people are likely to infer that the behavior is the result of a person’s attitudes, and conversely infer that situational factors are guiding behavior when external motivators are present. For example, people tend to attribute recycling behavior as being extrinsically motivated when others participate in financially compensated recycling programs, compared to when people participate in voluntary recycling programs [39]. This suggests that external motivators (e.g., financial compensation) make people attribute the behavior of others as extrinsically motivated. Similarly, people attribute other people’s lawful behavior to deterrence by the external motivator (the law), while their own behavior is attributed to being moral (internal motivator) [40], further suggesting that the same external motivator make people assume that others are extrinsically motivated, while themselves are intrinsically motivated.

There is reason to suspect that social norms may similarly be perceived as an external motivator when guiding other peoples’ behavior, but not necessarily as externally motivating when guiding an individual’s own behavior. According to Kelley’s covariation model [41], behavior that is high in consensus (e.g., social norms) signals that there is an external cause for the behavior, and while both descriptive and injunctive norms have been conceptualized and suggested as a form of external motivator [11, 42], other research show that the influence of descriptive norms on behavior is under-detected [43, 44], and thus may not be *perceived* as an external motivator, presumably in part as the process of conformity to descriptive norms can be automatic [45, 46]. However, this perception is asymmetrical, in that people assume that other people will be more influenced by a norm than they themselves will be [47]. This bias may partly stem from an asymmetrical access to introspective information, in that people have access to introspective knowledge about their own behaviors and thoughts, but have very limited knowledge about why other people behave the way they do [48]. Thus, social norms may be perceived as an external incentive for other people but not for oneself, and thus signal that other people are conforming to the norm due to an extrinsic motivation (e.g., due to social pressure). In this paper, we test if 1) information about others’ extrinsic or intrinsic motivation to conform could be experimentally manipulated, and 2) examine the consequences of extrinsically vs. intrinsically attributed norms on pro-environmental purchase intention.

### Hypotheses of attributing conformity extrinsically vs intrinsically

#### Conformity to specific norm

Indirect evidence that intrinsically motivated behavior could promote conformity comes from research showing that people conform more to others’ behavior when the behavior is consistent [15]. Consistent behavior is in turn more likely to be attributed to internal and invariant properties of the individual or group performing the behavior [41]. In contrast, the influence of social norms diminishes when people perceive that others are “sheepishly” following a leader, i.e., when perceiving that people are conforming without a clear cause [16]. Thus, although attribution of motivation may not have been directly studied in relation to social norm conformity, the above cited research suggest that such internal attributions may in fact increase conformity. However, as both the intrinsic and extrinsic norm condition will manipulate the same descriptive pro-environmental norm, we hypothesize that *(H1)*: *Both the intrinsic- and the extrinsic norm condition will increase conformity to a specific norm*, *compared to the control condition*. Based on the reasoning above, we also hypothesize that *(H2)*: *The intrinsic norm condition will increase conformity to a specific norm*, *compared to the extrinsic norm condition*.

#### Conformity to generalized norm

If the intrinsic norm condition increases conformity for one pro-environmental purchase intention, there may be reason to expect that conformity will also generalize to related pro-environmental intentions. Conformity to social norms can sometimes generalize to non-targeted, but related, behaviors, such that conformity to an initial pro-social behavior has been shown to increase the frequency of other pro-social behaviors not targeted by the initial norm manipulation [49]. While non-generalizing conformity is assumed by some to reflect imitation of behavior, generalizing conformity is assumed to happen when people understand the underlying motives of others [50]. Providing information that others are intrinsically motivated to perform a pro-environmental behavior may thus generalized more readily to related behaviors. Furthermore, as Kelley’s [41] covariation model suggest that people attribute consistent behavior to dispositional characteristics, they may also be able to infer that people whose behavior is caused by dispositional characteristics, such as intrinsic motivation, will also perform behaviors consistently, e.g., perform related behaviors. Similarly, it is often easier to predict future behavior from past behavior that is internally attributed, since internal attributions suggests that a person behaves according to their values, moral and motivations [51]. Thus, the manipulation of an intrinsic social norm may result in a perception that there is a norm for related pro-environmental behaviors as well, since the intrinsic attribution would suggest that people perform behaviors consistently and according to their values. We therefore hypothesize that *(H3)*: *The intrinsic norm condition will increase perception of other people’s conformity to a generalized norm*, *compared to the extrinsic norm condition*, and *(H4)*: *the control condition*. If the intrinsic norm increases peoples’ perception that there is a social norm for related behaviors, this generalized pro-environmental norm might in turn result in conformity to the related normative behavior *(H5)*: *The intrinsic norm condition will increase conformity to a generalized norm*, *compared to the extrinsic norm condition*, and *(H6)*: *the control condition*.

#### Overview of the studies

In two studies, we will investigate how attributing other peoples’ motivation to either intrinsic or extrinsic causes affects the influence of pro-environmental social norms. Specifically, we will test if information that other people are performing a normative behavior due to intrinsic motivation is more influential than if the behavior is performed due to extrinsic motivation. In Study 1, we study the effects of intrinsic vs extrinsic social norms on both specific and generalized environmentally friendly products. Study 2 replicates the specific and generalized effects using different products, and compares the intrinsic- and extrinsic norms to an injunctive norm condition.

## Study 1

The aim of Study 1 is to investigate whether a descriptive norm perceived to be intrinsically motivated increases conformity more than a descriptive norm perceived to be extrinsically motivated and a control condition. We will further test whether an intrinsic norm about pro-environmental behavior strengthens a belief that the people conforming to the manipulated norm also perform related pro-environmental behaviors, compared to an extrinsic norm and control condition. Additionally, we will test whether this perception of norms for related behaviors in turn results in greater conformity to the pro-environmental behavior. The design, hypotheses and analysis plan for Study 1 was pre-registered and can be found at: (https://osf.io/rv96b/?view_only=2d14e9683cb343fc9e3f45a888ab9aef)

### Method

The study followed ethical guidelines in Sweden for survey data and was thus conducted in line with the declaration of Helsinki, and in accordance with applicable laws governing research with human participants in Sweden. Participants gave written informed consent before participating and were debriefed after the study.

#### Pilot

To evaluate the manipulation, we ran a pilot-study on Amazons’ Mturk including 253 participants (control, *n* = 89, intrinsic norm, *n* = 83, extrinsic norm, *n* = 81). All participants were firstly informed that the survey investigated Mturkers’ view of eco-labeled chocolate and Mturkers’ product preferences, and that they would be presented with two similar products and asked a few questions about eco-labeling. The two norm conditions, intrinsic and extrinsic descriptive social norms, were induced by providing participants with fictive information about other Mturkers’ motivation. In the intrinsic norm condition, participants were informed that “*Mturkers’ taking our HITs have stated that the reasons for choosing the eco-labeled chocolate bar are because they feel a sense of pleasure when improving environmental quality and because they like the way they feel when doing things for the environment*”. In the extrinsic norm condition, participants were informed that “*Mturkers taking our HITs have stated that the reasons for choosing the eco-labeled chocolate bar are because of the recognition they get from others when doing things for the environment and because they want to avoid criticism*”. The wording from the two norm conditions was adapted from items for intrinsic and extrinsic motivation in the MTES scale [36]. In both norm conditions participants viewed the same communal shopping cart that contained the total product choice of Mturkers who had presumably taken our previous HITs, manipulating the descriptive norm for eco-labeled chocolate bars (80% choosing the eco-labeled option). The control condition was not exposed to the pro-environmental descriptive norm. The attention check suggested that 13.9% of participants incorrectly perceived the descriptive norm manipulation to be a minority norm. As a manipulation check of the perceived extrinsic and intrinsic motivation of others, participants also indicated how strongly they agreed with eight statements adapted from the MTES scale. The statements were preceded with the question “*If Mturkers taking our HITs were to choose the eco-labeled chocolate bar*, *what do you think their reason for doing so would be*?”, with responses ranging from 1 –*strongly disagree* to 7 –*strongly agree*. The statements included in the extrinsic scale were: *Because they want to avoid criticism*, *Because of the recognition they get from others*, *Because they want to avoid upsetting others*, and *Because their friends insist on it*. The statements for the intrinsic scale were: *Because they feel a sense of pleasure when improving environmental quality*, *Because they like the way it makes them feel*, *Because of the pleasure they feel when mastering new ways of helping the environment*, and *Because of the pleasure they feel when contributing to the environment*. Results confirmed that the intrinsic norm condition strengthened the belief that other participants were intrinsically motivated to conform to the pro-environmental norm, compared to the extrinsic norm condition, *t*(162) = 3.84, *p* < .001, *d* = 0.60 CI[.29, .91]. The extrinsic norm condition strengthened the belief that other participants were extrinsically motivated to conform to the pro-environmental norm compared to the intrinsic norm condition, *t*(162) = -4.69, *p* < .001, *d* = 0.73 CI[.42, 1.05]. The scales to measure intrinsic (Cronbach’s α = .85) and extrinsic motivation (Cronbach’s α = .88) showed acceptable reliability.

#### Participants

An a priori power calculation for binary logistic regression indicated that 644 was a sufficient sample size to detect an effect size of *OR* = 1.72 with greater than .80 power (α = .05). The effect size for our power calculation on is based on the meta-analytic effect size for implicit norms vs control conditions (*d* = 0.60, [3]), divided by half (i.e., *d* = 0.30), as we are also comparing two norm conditions which we assume result in a smaller effect. The pilot study indicated that approximately 14% of participants may not be attentive to the manipulation. To account for possible exclusions, we therefore recruited 735 participants on Mturk (*M*_age_ = 37.61, *SD* = 11.57, 62.6% male). Only MTurk users currently residing within the U.S, had a HIT approval rate of over 90%, had at least 100 HITs approved and were over 18 years were allowed to participate in the study. After excluding those who failed the attention check (see below), 684 participants (*M*_age_ = 37.89, *SD* = 11.54, 62% male) remained for the analyses.

#### Measures and procedures

The survey experiment employed a between-subjects design with one factor (type of social norm) and three conditions (control vs intrinsic norm vs extrinsic norm). The survey was created in Qualtrics and distributed via Mturk where each participant was paid $0.30 for their participation. The study was conducted in line with the declaration of Helsinki. Participants were informed that their data would be treated confidentially and used for research purposes only, and that they had the right to end their participation at any time. Each participant was randomly assigned to one of the three experimental conditions. All participants were firstly informed that the survey investigated Mturkers’ view of eco-labeled chocolate and Mturkers’ product preferences, and that they would be presented with two similar products and be asked to choose the one they would buy. Participants assigned to the control condition did not receive any additional information. The manipulation of the descriptive norm and Mturkers’ motivation was the same as in the pilot-study.

The primary dependent variable was conformity to the social norm in favor of the eco-labeled chocolate bar, which was measured as a binary choice between the eco-labeled chocolate bar and the regular chocolate bar. The stated price for the products was: regular chocolate bar = $2, eco-labeled chocolate bar = $4. To gauge whether the social norm in favor of an eco-labeled product would generalize to other pro-environmental products, participants were then presented with an energy star-labeled light bulb and a regular light bulb and asked to choose the product they think other Mturkers’ taking the HIT would buy (perception of others’ generalized conformity), as well as to choose the lightbulb they themselves would buy (generalized conformity). The order of the variables *perception of others’ generalized conformity* and *generalized conformity* was randomized. The stated price for the products was: regular light bulb = $5, energy-star labeled light bulb = $10.

As a general attention check, participants were then asked to indicate the correct cocoa percentage of the two chocolate bars, with response options being 30% or 70%. As a manipulation check of the perceived extrinsic and intrinsic motivation of others, participants also indicated how strongly they agreed with the eight statements adapted from the MTES scale (intrinsic motivation = Cronbach’s α = .84, extrinsic motivation = Cronbach’s α = .90). Lastly, participants indicated their age and gender and were debriefed.

### Results

#### Manipulation checks

Validating the manipulation, the intrinsic social norm increased intrinsic motivation compared to the control condition *t*(454) = 3.31, *p* = .001, *d* = 0.30 CI[0.13, 0.50], and compared to the extrinsic social norm *t*(454) = 3.92, *p* < .001, *d* = 0.36 CI[0.18, 0.55]. The extrinsic social norm significantly increased extrinsic motivation compared to the intrinsic social norm *t*(454) = -5.70, *p* < .001, *d* = 0.53 CI[0.35, 0.72], and compared to the control condition, *t*(454) = 7.06, *p* < .001, *d* = 0.66 CI[0.47, 0.85]. There was no significant difference in extrinsic motivation between the intrinsic norm condition and the control condition, *t*(454) = 1.30, *p* = .134, *d* = 0.12 CI[-0.06, 0.31], and there was no significant difference in intrinsic motivation between the extrinsic norm condition and the control condition, *t*(454) = -0.47, *p* = .642, *d* = 0.04 CI[-0.14, 0.23].

#### Main analyses

To test our hypotheses (see Table 1 for descriptive statistics and Table 2 for correlations), we conducted four binary logistic regressions with experimental condition as the sole IV and *own choice of chocolate bar* as DV for test of conformity to specific norm, *perception of others’ choice of light bulb* as DV for test of others’ conformity to generalized norm, and *own choice of light bulb* as DV for test of conformity to generalized norm. To test H1, the control condition was used as the reference category, while for test of H2-H6, the intrinsic norm condition was used as the reference category.

**Table 1:** Descriptive summary *M (SD)* of manipulation checks and dependent variables by experimental conditions.

	*Experimental condition*		
*Variable*	Intrinsic norm condition (*n* = 228)	Extrinsic norm condition (*n* = 228)	Control condition (*n* = 228)
Intrinsic motivation	5.39 (1.04)	5.00 (1.12)	5.05 (1.19)
Extrinsic motivation	3.91 (1.54)	4.72 (1.50)	3.72 (1.52)
Conformity to specific norm–percentage of eco-labeled choice	42.5%	36%	32.5%
Perception of others conformity to generalized norm–percentage of energy-star labeled choice	65.8%	64%	52.2%
Conformity to generalized norm—percentage of energy-star labeled choice	66.7%	62.3%	60.5%

**Table 2:** Correlation table with all measured variables.

	1. Intrinsic motivation	2. Extrinsic motivation	3. Conformity to specific norm (1 = eco-label)	4. Others’ conformity to generalized norm (1 = energy-star)	5. Conformity to generalized norm (1 = energy-star)	6. Age	7. Gender (female = 1)
1.		.096 (.009)	.193 (< .001)	.072 (.051)	.104 (.005)	-.001 (.984)	.006 (.871)
2.			-.013 (.715)	-.005 (.896)	-.079 (.032)	-.167 (< .001)	-.041 (.263)
3.				.143 (< .001)	.220 (< .001)	-.111 (.003)	.013 (.732)
4.					.527 (< .001)	.013 (.732)	-.021 (.571)
5.						.031 (.397)	-.021 (.574)
6.							.132 (< .001)

*Note*: *p*-values are in parentheses.

**Fig 1:**
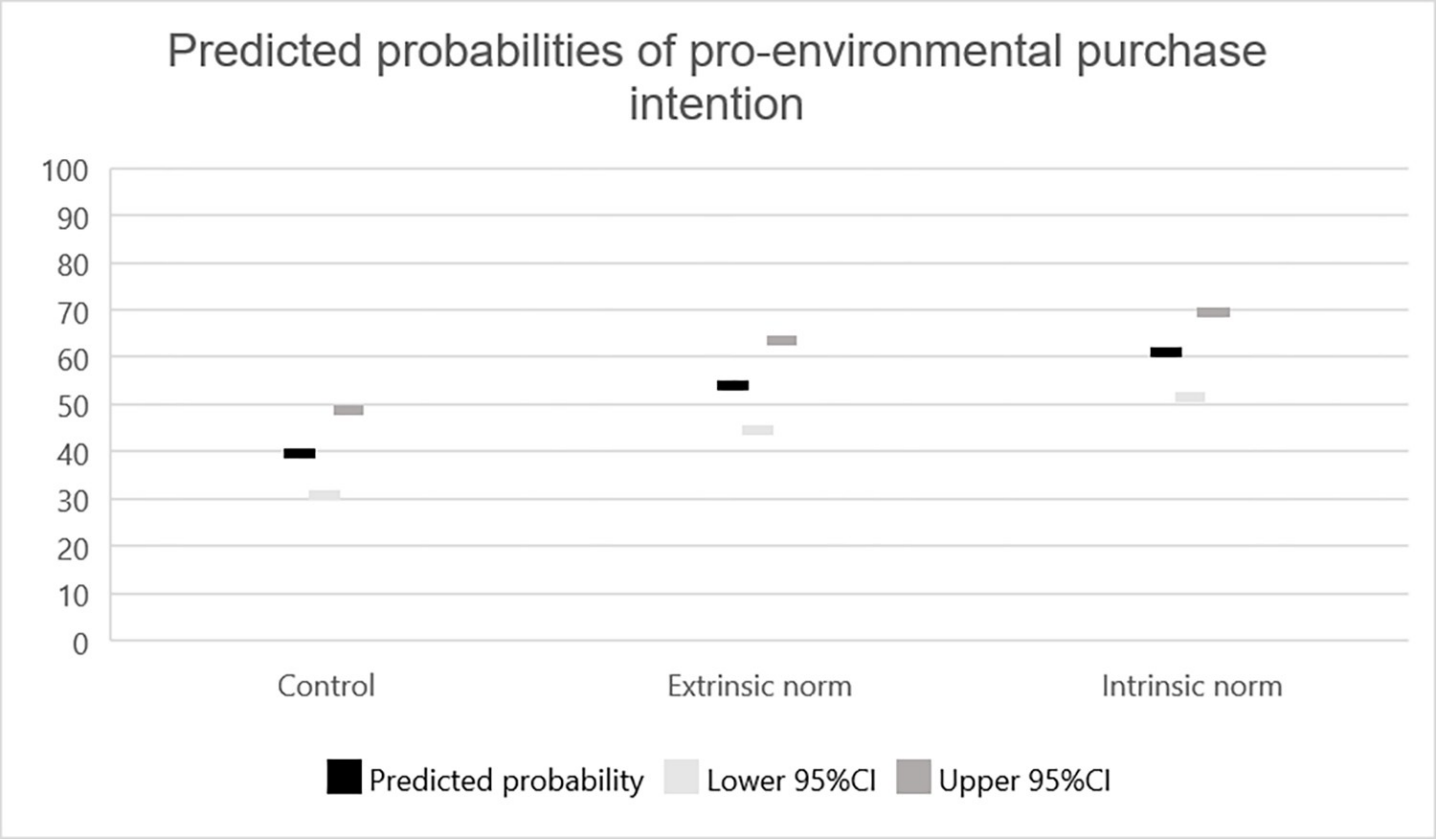
Predicted probabilities with 95% confidence intervals for conformity to specific norm (intention to purchase eco-labeled chocolate bar).

In partial support of H1, an intrinsic social norm increased conformity to the specific norm compared to the control condition, *b* = -.43(.20), *W* = 4.93, *p* = .026, *OR* = 0.65, CI[0.44, 0.95]. However, there was no significant difference between the extrinsic norm and the control condition, *b* = .16 (.20), *W* = .62, *p* = .430, *OR* = 1.17, CI[0.79, 1.72], or the intrinsic norm condition, *b* = -.28(.19), *W* = 2.07, *p* = .151, *OR* = 0.76, CI[0.52, 1.11], refuting H2 (see Fig 1). In support of H4, the intrinsic norm condition increased the belief of others’ conformity to a generalized norm compared to the control condition, *b* = -.57 (.19), *W* = 8.65, *p* = .003, *OR* = 0.57, CI[0.39, 0.83]. Refuting H3, there was no significant difference between the intrinsic and the extrinsic norm conditions, *b* = -.08 (.20), *W* = .15, *p* = .695, *OR* = 0.93, CI[0.63, 1.36]. There was no significant difference in conformity to a generalized norm between the intrinsic and extrinsic norm, *b* = -.19 (.20), *W* = .96, *p* = .328, *OR* = 0.83, CI[0.56, 1.21], or control condition, *b* = -.27 (.20), *W* = 1.85, *p* = .173, *OR* = 0.77, CI[0.52, 1.12], refuting H5 and H6.

#### Exploratory analysis

As the descriptive data suggested that an extrinsic norm might also increase the belief that others would conform to a generalized norm, we performed an additional, exploratory analysis with the control condition as the reference category. Results suggested that the extrinsic norm condition also increased the perceived conformity of others to a generalized norm, compared to control condition, *b* = .49 (.19), *W* = 6.53, *p* = .011, *OR* = 1.63, CI[1.12, 2.37].

Including inattentive participants in the analyses did not significantly affect the results.

### Discussion

In Study 1, we found that only the intrinsic norm increased pro-environmental conformity compared to the control condition. We did not observe the same effect for the extrinsic norm condition, indicating that the influence of normative information is dependent on attribution of the normative behavior. Perceiving that other people perform pro-environmental behaviors due to extrinsic motivation, in this case fear of social disapproval, does not appear to be a valid reason to adapt to the prescribed behavior. In contrast, perceiving that other people perform pro-environmental behaviors because they like the way they feel about themselves when improving environmental quality, i.e., are intrinsically motivated, appears to be a greater motivator to conform to pro-environmental behaviors. However, we do not know how this new attributional framing of norms stands in relation to more common forms of social norm framing. To address this, we will include an additional norm condition in Study 2, where an injunctive norm is manipulated in addition to the descriptive norm present in all norm conditions.

Concerning the generalizability of a pro-environmental norm to related pro-environmental behaviors, we saw that both the intrinsic and the extrinsic norm condition increased the perception that other participants would choose the pro-environmental option compared to the control condition, while there were no significant differences concerning participants’ own purchase intention of the pro-environmental option between any of the conditions. This pattern of results might either be due to the underlying motivation to conform not generalizing to the non-targeted behavior or to participants experiencing mixed motivations for choosing that specific product. Some participants might have reasoned that the energy-star light bulb is the cheaper option as it usually lasts longer than less energy efficient light bulbs. If participants were aware of this, they may also have expected people who were extrinsically motivated to choose the pro-environmental option simply to save money. As this dual motivation is a likely issue with most energy products, Study 2 will measure how norms generalize to products with no such motivational ambiguity, namely MSC-certified fish products. Additionally, the behavioral similarity between pro-environmental food products and energy products may be too weak for motivation to generalize effectively. In Study 2, we will therefore study the effects of generalization on more related products, namely on different pro-environmental food products.

The manipulation check indicated that while the intrinsic (extrinsic) norm condition increased the belief that other participants were intrinsically (extrinsically) motivated compared to the control condition, it did not decrease the belief that other participants were extrinsically (intrinsically) motivated compared to the control condition. To better separate the effects of extrinsic and intrinsic motivation and potentially increase the strength of the effects, the manipulation of motivation in Study 2 will additionally include a description that intrinsically (extrinsically) motivated individuals are not extrinsically (intrinsically) motivated. In an additional attempt to increase the power to detect any real effects, Study 2 will measure two dependent variables for each set of hypotheses and on continuous scales.

## Study 2

In this study, we will refine the manipulation of extrinsic and intrinsic motivation and additionally compare the positive effect of an intrinsic social norm to a common manipulation of social norms; injunctive norms. When manipulating injunctive norms, we typically provide information of what kind of behavior other people approve of or expect others to perform. As injunctive norms do not explicitly convey the motivation behind other peoples’ approval, this information could be interpreted to mean that either other people approve of a behavior due to extrinsic motivation (e.g., they gain from the behavior or are sensitive to social expectations) or out of intrinsic motivation (e.g., the behavior is in line with that groups’ attitudes or is expected to lead to a valued outcome). Thus, the motivation behind the injunctive norm condition may not be as uniformly interpreted as the intrinsic norm condition, potentially reducing the normative impact. We hypothesize that: *(H1) the intrinsic norm condition will increase conformity to a specific norm*, *compared to the extrinsic norm condition*, *(H2) the injunctive norm condition*, and *(H3) the control condition*. We further hypothesize that: *(H4) the intrinsic norm condition will increase a perception of other people’s conformity to a generalized norm*, *compared to the extrinsic norm condition*, *(H5) the injunctive norm condition* and *(H6) the control condition*, and that: *(H7) the intrinsic norm condition will also increase conformity to a generalized norm*, *compared to the extrinsic norm condition*, *(H8) the injunctive norm condition* and *(H9) the control condition*.

The design, hypotheses and analysis plan for Study 2 was pre-registered and can be found at: (https://osf.io/n35pm/?view_only=49095034cb9642a0ba5c5513ee7d70be).

### Method

The study followed ethical guidelines in Sweden for survey data and was thus conducted in line with the declaration of Helsinki, and in accordance with applicable laws governing research with human participants in Sweden. Participants gave written informed consent before participating and were debriefed after the study.

#### Participants

An a priori power calculation for MANOVA indicated that 572 was a sufficient sample size to detect an effect size of *f* = .12 with greater than .80 power (α = .05), when within measures are correlated at *r* = 0.5. The effect size we based our power calculation on is based on the effect size for the difference between the intrinsic norm condition and the control condition in Study 1. Study 1 suggested that approximately 7% of participants may not be attentive as indicated by an attention check. To account for this and the possibility of larger exclusions with the new attention check, we therefore recruited 700 participants on Mturk. Due to a technical error, the data of three participants could not be collected and the final sample was therefore 697 Mturk users (*M*_age_ = 38.25, *SD* = 12.19, 56.2% male). Only Mturk users currently residing within the U.S, had a HIT approval rate of over 90%, had at least 100 HITs approved, were over 18 years and had not participated in Study 1 were allowed to participate in the study. After excluding those who failed the attention check (see below), 642 participants (*M*_age_ = 38.17, *SD* = 12.16, 56% male) remained for the analyses.

#### Procedure and measures

The survey experiment employed a mixed subjects design with one factor (type of social norm) and four conditions (control vs injunctive norm vs intrinsic norm vs extrinsic norm), and two within measures (purchase intention). The survey was created in Qualtrics and distributed via Mturk where each participant was paid $0.40 for their participation. Each participant was randomly assigned to one of the four experimental conditions, and all were firstly informed that the survey investigated Mturkers’ view of organic products and their product preferences. The *control condition* did not receive any additional information. The *intrinsic norm* was manipulated by stating that “*Mturkers taking our HITs have stated that the reasons for choosing organic products are because they feel a sense of pleasure when improving environmental quality*, *and because they like the way they feel when doing things for the environment*. *They state that getting recognition from others when doing things for the environment and wanting to avoid criticism are not reasons for choosing organic products*”. The *extrinsic norm* was manipulated by declaring that “*Mturkers taking our HITs have stated that the reasons for choosing organic products are because of the recognition they get from others when doing things for the environment and because they want to avoid criticism*. *They state that feeling a sense of pleasure when improving environmental quality or liking the way they feel when doing things for the environment are not reasons for choosing organic products*”. Finally, the *injunctive norm condition* was manipulated by stating that “*Mturkers taking our HITs have stated that they approve of buying organic products and expect others to do so*”. In all three norm conditions participants viewed the same communal shopping cart that contained the total product choice of Mturkers who had presumably taken our previous HITs, manipulating the descriptive norm for organic milk (70% choosing the organic option) and organic coffee (80% choosing the organic option).

The two primary dependent variables were conformity to the social norm in favor of the organic coffee and milk, measured with the items “*Which coffee/milk would you buy*?”, on a scale ranging from 1 –*Definitely the regular coffee/milk* to 7 –*Definitely the organic coffee/milk*, presented in random order. The stated price for the products was: regular coffee = $3.99, organic coffee = $7.99, regular milk = $1.99, organic milk = $3.99. To gauge whether the social norm in favor of the organic products would generalize to other pro-environmental products, participants were then presented with both an MSC-certified tuna and salmon product and both a regular tuna and salmon product and asked to indicate the two products they think other Mturkers’ taking the HIT would buy (perception of others’ generalized conformity), as well as to indicate the two products they themselves would buy (generalized conformity). All four dependent variables were measured on a scale from 1 –*Definitely the regular tuna/salmon* to 7 –*Definitely the MSC-certified tuna/salmon*. The order of *perception of others’ generalized conformity* and *generalized conformity* was randomized, as was the order of the two product choices (tuna and salmon products). The stated price for the products was: regular tuna = $1.99, MSC-certified tuna = $3.99, regular salmon = $3.99, MSC-certified salmon = $7.99.

As a general attention check, participants were then asked to indicate which of the two milk products presented in the beginning of the survey was more expensive, with response options being the regular milk or the organic milk. Participants answered the same manipulation checks as in Study 1, with Cronbach’s α for the extrinsic motivation scale = .91, and Cronbach’s α for the intrinsic motivation scale = .88. Lastly, participants indicated their age and gender and were debriefed.

### Results

#### Manipulation checks

Validating the manipulation, the intrinsic social norm increased intrinsic motivation compared to the control condition *t*(315) = 4.91, *p* < .001, *d* = 0.55 CI[0.33, .078], compared to the extrinsic social norm *t*(279.05) = -7.06, *p* < .001, *d* = 0.78 CI[0.56, 1.02], and compared to the injunctive norm condition, *t*(314) = 3.46, *p* < .001, *d* = 0.38 CI[0.17, 0.61]. The extrinsic social norm significantly increased extrinsic motivation compared to the intrinsic social norm *t*(317) = 3.91, *p* < .001, *d* = 0.44 CI[0.22, 0.66], compared to the control condition, *t*(324) = 4.30, *p* < .001, *d* = 0.48 CI[0.26, 0.70], and compared to the injunctive norm condition, *t*(323) = 3.32, *p* = .001, *d* = 0.36 CI[0.15, 0.59]. There was no significant difference in extrinsic motivation between the intrinsic norm condition and the control condition, *t*(315) = .25, *p* = .804, *d* = 0.03 CI[-0.19, 0.25], or the injunctive norm condition, *t*(306.94) = -.82, *p* = .412, *d* = 0.10 CI[-0.13, 0.31]. The extrinsic norm condition significantly decreased intrinsic motivation compared to the control condition, *t*(324) = -3.01, *p* = .003, *d* = 0.34 CI[0.12, 0.55], and compared to the injunctive norm condition, *t*(281.54) = -4.36, *p* < .001, *d* = 0.49 CI[0.26, 0.70]. There was no significant difference between the injunctive norm condition and the control condition in intrinsic motivation, *t*(321) = -1.58, *p* = .116, *d* = 0.18 CI[-0.04, 0.39], or in extrinsic motivation, *t*(321) = -1.12, *p* = .263, *d* = 0.13 CI[-0.09, 0.34].

#### Main analyses

To analyze our hypotheses (see Table 3 for descriptive statistics and Table 4 for correlations), we conducted three (one for each group of dependent variables) MANOVAs. For the analyses of H1-H3 and H7-H9, the two MANOVAS were run with three dummy coded variables for the experimental conditions as IVs and the two product ratings as dependent variables. The intrinsic norm condition was coded as 0, and all other conditions were coded as 1 for one of the three dummy variables. As the dependent variables used to analyze H4-H6 did not meet the assumption of covariance homogeneity, as indicated by Box’s test (*p* = .035), this MANOVA was carried out in the R package MANOVA.RM, with *p*-values from multivariate Dunnett planned contrasts used as inference criteria, as specified by the pre-registered analysis plan. Performing a MANOVA that did not assume covariance homogeneity did not alter the interpretation of the results compared to a MANOVA that did assume multivariate homogeneity.

**Table 3:** Descriptive summary of *M (SD)* of manipulation checks and dependent variables by experimental conditions.

	*Experimental condition*			
*Variable*	Intrinsic norm condition (*n* = 155)	Injunctive norm condition (*n* = 161)	Extrinsic norm condition (*n* = 164)	Control condition (*n* = 162)
Intrinsic motivation	5.51 (1.01)	5.12 (1.03)	4.47 (1.58)	4.93 (1.11)
Extrinsic motivation	3.74 (1.63)	3.89 (1.46)	4.45 (1.61)	3.70 (1.55)
Conformity to specific norm—milk	4.26 (2.43)	4.01 (2.28)	3.49 (2.31)	3.46 (2.31)
Conformity to specific norm—coffee	3.60 (2.35)	3.76 (2.27)	3.32 (2.26)	3.25 (2.27)
Others’ conformity to generalized norm—tuna	4.90 (1.88)	5.04 (1.69)	4.49 (1.91)	3.59 (2.00)
Others’ conformity to generalized norm—salmon	4.95 (1.95)	5.32 (1.48)	4.55 (1.85)	3.71 (1.89)
Conformity to generalized norm—tuna	3.83 (2.39)	3.79 (2.16)	3.74 (2.23)	3.76 (2.31)
Conformity to generalized norm—salmon	3.92 (2.27)	4.25 (2.24)	3.74 (2.25)	3.75 (2.31)

**Table 4:** Correlation table of all measured variables in Study 2.

	1. Intrinsic motivation	2. Extrinsic motivation	3. Conformity to specific norm—milk	4. Conformity to specific norm—coffee	5. Perceived conformity to generalized norm—tuna	6. Perceived conformity to generalized norm—salmon	7. Conformity to generalized norm—tuna	8. Conformity to generalized norm—salmon	9. Age	10. Gender (female = 1)
1.		.013 (.742)	.286 (< .001)	.296 (< .001)	.209 (< .001)	.209 (< .001)	.264 (< .001)	.236 (< .001)	.040 (290)	.008 (830)
2.			.148 (< .001)	.234 (< .001)	.217 (< .001)	.232 (< .001)	.170 (< .001)	.120 (.001)	-.081 (.032)	-.095 (.012)
3.				.707 (< .001)	.351 (< .001)	.359 (< .001)	.660 (< .001)	.663 (< .001)	-.034 (.365)	.-.036 (347)
4.					.354 (< .001)	.413 (< .001)	.667 (< .001)	.684 (< .001)	-.098 (.010)	-.062 (.101)
5.						.729 (< .001)	.474 (< .001)	.378 (< .001)	-.018 (.634)	.021 (.573)
6.							.465 (< .001)	.483 (< .001)	-.014 (.710)	.081 (.032)
7.								.761 (< .001)	-.066 (.083)	.009 (815)
8.									-.012 (.751)	.023 (.552)
9.										.104 (.006)

*Note*: *p*-values are in parentheses.

**Fig 2:**
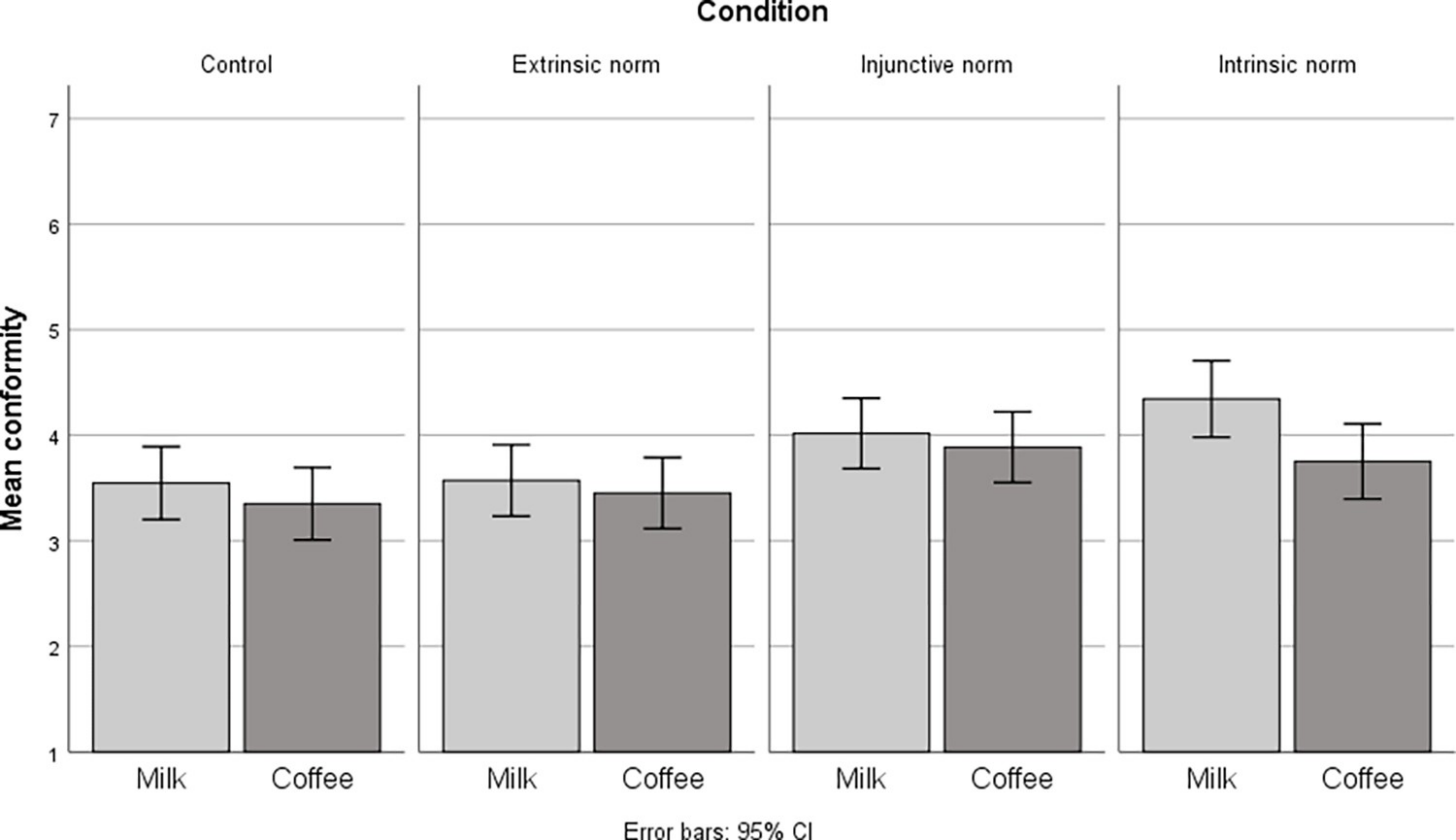
Means with 95% confidence interval for conformity to specific norm (intention to purchase organic milk and coffee products presented separately).

*Conformity to specific norm*. In support of H1, an intrinsic social norm significantly increased conformity to a specific norm compared to an extrinsic social norm, *V* = .02, *F*(2, 637) = 5.25, *p* = .005, *η*_p_^2^ = .02 CI[.00, .03]. Refuting H2, there was no significant difference between the intrinsic social norm and the injunctive social norm, *V* = .01, *F*(2, 637) = 2.19, *p* = .113, *η*_p_^2^ = .01 CI[.00, .02]. In support of H3, the intrinsic social norm significantly increased conformity to a specific norm compared to the control condition, *V* = .02, *F*(2, 637) = 5.19, *p* = .006, *η*_p_^2^ = .02 CI[.00, .03] (see Fig 2).

*Perceived conformity to generalized norm*. Refuting H4 and H5, there was no significant difference between an intrinsic social norm and an extrinsic social norm, *MD* = -0.82 CI[-1.99, 0.36], *p* = .219, or an injunctive norm, *MD* = 0.50 CI[-0.60, 1.60], *p* = .533. In support of H6, an intrinsic social norm significantly increased a perception that other Mturkers would conform to a generalized norm, compared to the control condition, *MD* = -2.56 CI[-3.76, -1.36], *p* < 001.

*Conformity to generalized norm*. Refuting H7, H8 and H9, an intrinsic social norm did not increase conformity to a generalized norm compared to the extrinsic social norm, *V* = .00, *F*(2, 637) = .30, *p* = .741, η *η*_p_^2^ = .00 CI[.00, .01], the injunctive norm, *V* = .01, *F*(2, 637) = 2.47, *p* = .086, *η*_p_^2^ = .01 CI[.00, .02], or the control condition, *V* = .00, *F*(2, 637) = .29, *p* = .747, *η*_p_^2^ = .00 CI[.00, .01].

#### Exploratory analysis

*Perceived conformity to generalized norm*. As the descriptive data suggested that all three norm conditions may have increased a perception that other Mturkers would conform to a generalized norm, we performed an additional MANOVA in R as an exploratory analysis with the control condition as the reference category. Results suggested that, in addition to the intrinsic social norm, the extrinsic social norm increased a perception that other participants would conform to a generalized norm, *MD* = 1.74 CI[0.55, 2.93], *p* = .004, as well as the injunctive norm, *MD* = 3.06 CI[1.94, 4.17], *p* < 001.

*Conformity to specific norm*. As the descriptive data further suggested that an injunctive norm might also increase conformity to the specific norm we performed an additional exploratory MANOVA, with the control group as the reference category. Results suggested that an injunctive norm did not increase conformity to the specific norm compared to the control condition, *V* = .01, *F*(2, 637) = 2.50, *p* = .083, *η*_p_^2^ = .01 CI[.00, .02], nor did the extrinsic norm, *V* = .00, *F*(2, 637) = .05, *p* = .948, *η*_p_^2^ = .00 CI[.00, .00].

The inclusion of inattentive participants did not overall affect the results, except that the intrinsic norm now also increased conformity to a specific norm compared to the injunctive norm condition, *V* = .01, *F*(2, 692) = 3.05, *p* = .048, *η*_p_^2^ = .01 CI[.00, .02], while the injunctive norm increased conformity to a generalized norm, compared to the control condition, *V* = .01, *F*(2, 692) = 5.03, *p* = .007, *η*_p_^2^ = .01 CI[.00, .03].

### Discussion

In Study 2 we again observed that an intrinsic norm increased conformity compared to the control condition. In Study 2 we also found that an intrinsic norm increased conformity relative to an extrinsic norm, while neither the extrinsic norm nor the injunctive norm significantly increased conformity compared to the control condition. It appears that it is mainly the increase in intrinsic motivation that has a positive effect on conformity, and not the absence of extrinsic motivation, as the intrinsic norm condition and the injunctive norm condition did not significantly differ in mean extrinsic motivation. Supplementing the manipulation with information that extrinsically (intrinsically) motivated people were not intrinsically (extrinsically) motivated successfully decreased intrinsic motivation for the extrinsic norm condition compared with the control condition. However, it did not successfully decrease extrinsic motivation in the intrinsic norm condition, compared to the control. This may reflect that extrinsic motivation is perceived to be a more common motivator for people in a norm context, and therefore more difficult to manipulate. An injunctive norm appears to not significantly affect the perception of either the extrinsic or intrinsic motivation of others, as indicated by the fact that it was not significantly different from the control condition. In line with our theoretical reasoning that a perception that others are performing behaviors because they are intrinsically motivated should increase conformity, the injunctive norm condition did not increase conformity relative to the control condition. Descriptively, the injunctive norm however appears to affect conformity similarly as the intrinsic social norm, albeit slightly weaker. This suggests that aligning descriptive and injunctive norms does not significantly affect attributional processes, but rather may have a positive influence on conformity mainly due to its’ normative influence. While the assumed motivational basis for injunctive norms is to seek (avoid) social (dis)approval, our data suggest that this is not how it is mainly perceived to motivate other people.

As in Study 1, all three norm conditions increased the perception that other people will conform to a generalized norm, while we observed no significant differences between any of the conditions regarding peoples’ own conformity to a generalized norm. This suggests that the results of Study 1 were not due to energy efficient products allowing for ambiguous motivational attributions, but that people assume that other peoples’ normative behavior can be readily generalized to related behaviors regardless of underlying motivation, while their own conformity does not generalize to related purchase intentions.

## General discussion

In two experiments, we provided evidence that intrinsic norms (i.e., descriptive norms informing that others are intrinsically motivated) increase pro-environmental purchase intentions compared to both extrinsic norms (i.e., descriptive norms informing that others are extrinsically motivated) (Exp. 2) and a no information control condition (Exp. 1 and 2). These results are in line with our proposition that the influence of descriptive norm can be strengthened by framing the behavior as intrinsically motivated. That is, communicating that the behavior is valued and perceived as important to the people performing the behavior. This could in itself signal to the individual that conforming to the norm will effectively produce an outcome that is valued by the group, in line with how other types of internalized norms, such as personal norms, motivate conformity [11, 22, 23]. We did not observe that an extrinsic norm resulted in a decrease in conformity relative to the control condition in either of the studies, suggesting that while extrinsically motivated behavior may not be effective in changing peoples’ pro-environmental purchase intentions, it also does not have a negative effect on it. The lack of a positive effect of the extrinsic norm may however lend some insight as to why norm-based interventions are not always successful [52–54]. If the norm manipulation itself or the targeted behavior can signal an external motivation (e.g., norm interventions aimed at reducing energy usage may inadvertently signal that others’ save energy to save money, while at other times norm interventions may simply signal that others’ perform a common behavior mindlessly), it can potentially reduce the impact of the intervention. For example, adding a contest to a norm intervention has been shown to undermine the influence of the norm [55], suggesting that certain norm interventions may fail if the descriptive norm is attributed to extrinsic motivation, e.g., if people perceive that others are performing a normative behavior to win a contest.

There was no significant difference between an intrinsic norm and an injunctive norm (when analyzing attentive participants only), though only the intrinsic norm increased conformity compared to the control condition. While the lack of a significant difference between the injunctive norm and control condition may be a power-issue, the results indicate that providing information that people are intrinsically motivated to perform pro-environmental behaviors is still relatively more effective than providing information that other people approve of pro-environmental behaviors and expect others to perform them. As reasoned about norm interventions in general, the positive effect typically observed of injunctive norms may to an extent be dependent on how the norm is worded. For example, words suggesting that other people expect a certain behavior may cause the norm itself to be perceived more as an external incentive, potentially limiting the positive impact, while wordings such as “others approve” may make the norm be perceived as less of an external motivator. Similarly, the positive impact we typically see from injunctive norms could potentially be restrained by some individuals who experience reactance to the behavioral expectations communicated by the injunctive norm. Furthermore, the effect of injunctive norms could also depend on how intrinsically vs extrinsically motivated the specific sample is, as people may potentially be more likely to infer that other people are intrinsically motivated if they themselves are intrinsically motivated, in line with a false consensus bias [56].

Although participants perceived that other people would conform to a generalized norm irrespective of norm condition, none of the norms increased conformity to the generalized norm. As the intrinsic norm did not significantly increase the perception that others would conform to a generalized norm compared to the extrinsic norm or the injunctive norm, we should not, based on our theoretical reasoning, expect that the intrinsic norm would increase conformity to a generalized norm. The results suggest that the motivation underlying conformity to the norm did not generalize to the new behavior, but rather that only the pro-environmental descriptive norm did. As we observed that the intrinsic motivation of others was vital for peoples’ conformity to the specific norm, we should not expect them to conform to a generalized descriptive norm unless they perceive that other people are also intrinsically motivated to perform the new behavior. This further indicates that other peoples’ motivation is important for one’s own conformity but not for the perception of other peoples’ conformity, potentially lending further insight to the asymmetrical perception of social influence [47].

### Limitations

While the data pattern supports the interpretation that lack of conformity to the generalized norm was due to the underlying motivation not generalizing across behaviors, we did not actually measure perceived motivation for the generalized norm and thus cannot draw any firm conclusions as to the lack of effect. Furthermore, a limitation with these two studies is that we did not measure the assumed process of why intrinsically motivated norms positively affect conformity. While we argue that this attribution of norms would make the behavior appear more important and valued by the group, hence making the descriptive norm more valid and conformity more effective in producing a valued outcome, this was not confirmed nor were other explanations ruled out. Although we have demonstrated that these effects hold for a range of pro-environmental purchase intentions, future research should replicate these effects with actual behaviors, as people might experience different tradeoffs when having to spend actual money for pro-environmental products.

### Implications and future research

The results suggest that when aiming to use social norms to influence peoples’ pro-environmental purchase intentions, the effectiveness of the interventions can be improved by communicating that a certain behavior or attitude is the expression of an intrinsic motivation, rather than, or in addition to, relying on the commonly used alignment between injunctive and descriptive norms. Highlighting that other people are intrinsically motivated may be especially relevant in situations where there are already additional external incentives in place (e.g., governmental subsidies), as people in those situations may be more likely to infer that people in favor of the behavior are extrinsically motivated, potentially decreasing the effectiveness of the intervention. Future research might also consider whether certain behaviors themselves can affect how people attribute motivation, e.g., might behavior perceived to be autonomic or the result of a habit be perceived as a motivated or relatively less intrinsically motivated? Furthermore, these studies employed a presumed in-group as the reference group (other MTurkers). The positive effect of an intrinsic norm might be stronger when applied to a person’s outgroup, as evidence indicate that people are more likely to attribute positive outgroup behavior to external events [57]. Similarly, the general tendency of people to conform more to in-group norms than to outgroup norms or general norms may in part be related to the tendency to attribute positive in-group behavior to internal factors [58]. This is something future research might consider.

Concerning the strength of these effects, even though the effect size of the effectiveness of an intrinsic social norm compared to an extrinsic norm in Study 2 may not appear that large (*d* = .29), the effect sizes of the manipulation checks between the two conditions were also relatively small (intrinsic motivation *d* = .78, extrinsic motivation *d* = .44), when compared to the mean manipulation check effect size in social psychology (*d* = 1.58, [59]). Taking the mean of these two manipulation checks, the effect size (of the DV) is roughly half of the cause size (effect size of the MC), which can be compared to the median causal efficacy in social psychological research which is roughly one-third, indicating that the relationship between the manipulation and dependent variable is of medium to high strength [59]. Thus, these effects could potentially be quite impactful with a stronger manipulation and in situations where people can draw strong conclusions as to the intrinsic or extrinsic motivation of other people.

## Conclusion

Peoples’ beliefs about *why* other people are performing a pro-environmental behavior is vital for the conformity to social norms. Perceiving that other people perform pro-environmental behaviors due to dispositional characteristics, such as intrinsic motivation, increases conformity relative to perceiving pro-environmental behavior to be the result of situational factors, such as extrinsic motivation. In two studies we showed that intrinsic descriptive norms are more effective in promoting pro-environmental purchase intentions than extrinsic descriptive norms.
